# PtNi Alloy Coated in Porous Nitrogen-Doped Carbon as Highly Efficient Catalysts for Hydrogen Evolution Reactions

**DOI:** 10.3390/molecules27020499

**Published:** 2022-01-14

**Authors:** Xuyan Song, Yunlu He, Bo Wang, Sanwen Peng, Lin Tong, Qiang Liu, Jun Yu, Haolin Tang

**Affiliations:** 1 China Tobacco Hubei Industrial Co., Ltd., Wuhan 430051, China; songxy@hbtobacco.cn (X.S.); heyunlu@hbtobacco.cn (Y.H.); wangbo@hbtobacco.cn (B.W.); tonglin@hbtobacco.cn (L.T.); liuqiang@market.hbtobacco.cn (Q.L.); 2 China Tobacco Hubei Industrial Cigarette Materials Co., Ltd., Wuhan 430051, China; pengsw@market.hbtobacco.cn; 3 State Key Laboratory of Advanced Technology for Materials Synthesis and Processing, Wuhan University of Technology, Wuhan 430070, China

**Keywords:** hydrogen evolution reaction, porous carbon, PtNi alloy

## Abstract

The development of low platinum loading hydrogen evolution reaction (HER) catalysts with high activity and stability is of great significance to the practical application of hydrogen energy. This paper reports a simple method to synthesize a highly efficient HER catalyst through coating a highly dispersed PtNi alloy on porous nitrogen-doped carbon (MNC) derived from the zeolite imidazolate skeleton. The catalyst is characterized and analyzed by physical characterization methods, such as XRD, SEM, TEM, BET, XPS, and LSV, EIS, it, v-t, etc. The optimized sample exhibits an overpotential of only 26 mV at a current density of 10 mA cm^−2^, outperforming commercial 20 wt% Pt/C (33 mV). The synthesized catalyst shows a relatively fast HER kinetics as evidenced by the small Tafel slope of 21.5 mV dec^−1^ due to the small charge transfer resistance, the alloying effect between Pt and Ni, and the interaction between PtNi alloy and carrier.

## 1. Introduction

Hydrogen has the advantages of having high energy density and being clean and pollution-free, making it an ideal energy source to replace traditional fossil fuels to solve environmental and energy issues [1,2,3]. The development of an environmentally sound and sustainable hydrogen production method is the basis for the application of hydrogen energy. Hydrogen production by electrolysis of water has received extensive attention due to its clean and environmental advantages. The hydrogen evolution reaction is the key reaction to produce hydrogen through water electrolysis. To date, platinum (Pt) and Pt-based catalysts are still the ideal catalysts for the hydrogen evolution reaction because of their high catalytic activity and long-term stability. Unfortunately, the high cost and limited reserves hinder its large-scale promotion and commercial use.

Improving the catalytic activity and utilization rate of precious platinum to reduce the usage of platinum has become the key measure to break this deadlock. Supported platinum-based alloys with a high specific surface area is a research focus in reducing the content of platinum in the catalyst and improving the catalytic activity of the catalyst [4,5]. Benefiting from the synergistic effect induced by the interaction of Pt and transition metals, the combination of Pt with transition metals (Fe, Co, Ni, etc.) to form a composite alloy is an effective way to optimize the utilization of Pt and to improve its electrocatalytic activity [6,7]. As an example, Huang et al. [8] synthesized PtNi nanodentrites (PtNi NDs) by a simple solvothermal method. The transition metal Ni can adjust the inherent electronic structure of Pt to improve the catalytic activity. In 0.5 M H_2_SO_4_, the optimal sample PtNi NDs requires only 22 mV overpotential under current density of 10 mA cm^−2^, better than commercial 20 wt% Pt/C (30 mV) under the same condition. The resulting Tafel slope of 52 mV dec ^−1^ is much lower than that of 20 wt% Pt/C (66 mV dec^−1^).

Metal-organic framework (MOFs) materials have attracted great attention in the field of catalysis due to their high specific surface area and easy regulation of pore size and coordination center of metal atoms [9]. Meanwhile, derivatives of MOFs materials as the carrier materials of Pt-based catalysts can greatly improve the utilization of the precious metal Pt, thereby achieving the purpose of reducing the amount of the precious metal. As a branch of MOFs material, ZIFs (zeolite imidazolium ester skeleton structure materials) is widely used in the field of electrolytic water catalysis [10,11]. For example, Qin et al. [12] synthesized the PtCo bimetallic catalyst with ZIF-67 as a porous carbon source through the simple impregnation method. The Pt content of the optimal sample CPt@ZIF-67-900-6 is only 5 wt%. When the current density reaches 10 mA cm^−2^, the overpotential of CPt@ZIF-67-900-6(50 mV) is 5 mV lower than that of commercial 20 wt% Pt/C and the Tafel slope of CPt@ZIF-67-900-6 (27.1 mV dec^−1^) is much lower than that of the 20 wt%Pt/C catalyst (35.5 mV dec^−1^). The density functional theory calculation proves that ZIFs derivatives can improve the utilization rate of Pt.

Inspired by the above-mentioned analysis, porous carbon (MNC) support is prepared in this paper by the carbonization of Co-doped ZIF-8. The thus-obtained porous carbon not only has the advantage of a large specific surface area, but also contains a large number of Co-N_x_ active sites, which can enhance the catalytic activity [13,14]. Then, the electrocatalyst PtNi-MNC-x-y (x:y is the mass ratio of Co and Zn) is prepared by supporting platinum-nickel alloy on the porous carbon through a simple impregnation method. The effect of synthetic conditions on the microstructure and performance of materials is discussed in detail. The results revealed that the sample derived from a carbonization temperature of 900 °C and Co:Zn of 1:9 displayed the best performance due to the small charge transfer resistance, the alloying effect between Pt and Ni, and the interaction between PtNi alloy and the carrier.

The experimental details and electrochemical measurements are described in the Supplementary Material.

## 2. Results and Discussion

### 2.1. Material Characterization

**Figure 1:**
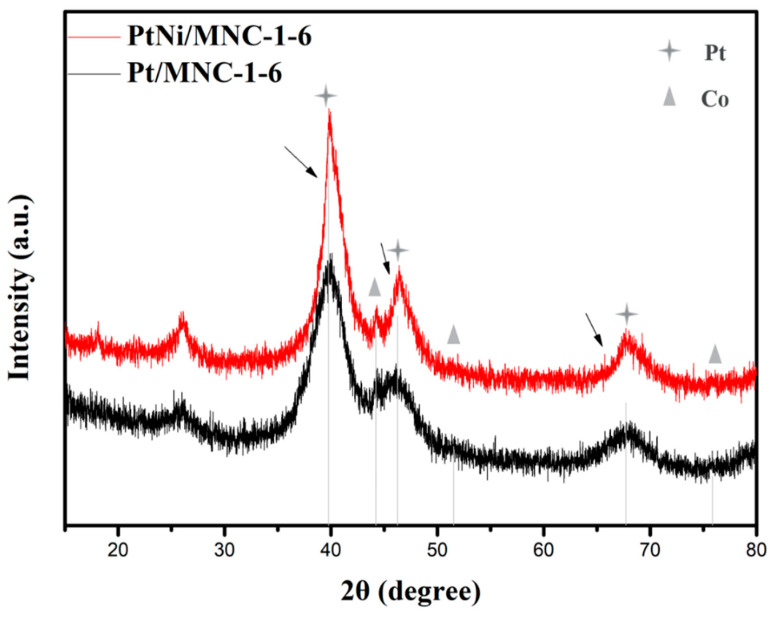
XRD patterns of PtNi/MNC-1-6 and Pt/MNC-1-6.

X-ray diffraction technology was first applied to analyze the crystal structure of the sample. Figure 1 shows XRD patterns of PtNi/MNC-1-6 and Pt/MNC-1-6. Although the Zn element was added during the materials preparation process, it can be almost completely removed as confirmed by the XRD patterns and the ICP-AES results (Table 1). In Figure 1, it can be observed that there are three diffraction peaks at 39.8°, 46.2° and 67.7°, corresponding to crystal planes of Pt (111), Pt (200), and Pt (220), respectively (ICDD 04-0802). Moreover, the observed slightly positive shift of Pt diffraction peaks (as shown by the arrow in the Figure 1) for PtNi/MNC-1-6 compared to the sample of Pt/MNC-1-6 suggested the change of lattice spacing of Pt after the formation of the PtNi alloy, confirming the formation of the PtNi alloy. In addition, three diffraction peaks of Co element can also be observed at 44.2°, 51.5° and 75.9° corresponding to Co (111), Co (200) and Co (220) crystal planes (ICDD 15-0806), respectively. A certain amount of Co–N_x_ can be formed during the pyrolysis process, which has been proved to be the active site to the electrolytic reaction [15]. The position of Co diffraction peaks has no obvious shift from the peak of the standard metal Co, indicating that the Co element does not form an alloy with Pt or Ni metal. Moreover, there is no obvious diffraction peak of nickel, which is speculated to be caused by the low content of nickel element (2.9 wt % as shown in Table 1). Additionally, Co (ICDD 15-0806) and Ni (ICDD 04-0850) have similar peak positions, which made it difficult to show the diffraction peak of nickel clearly.

**Figure 2:**
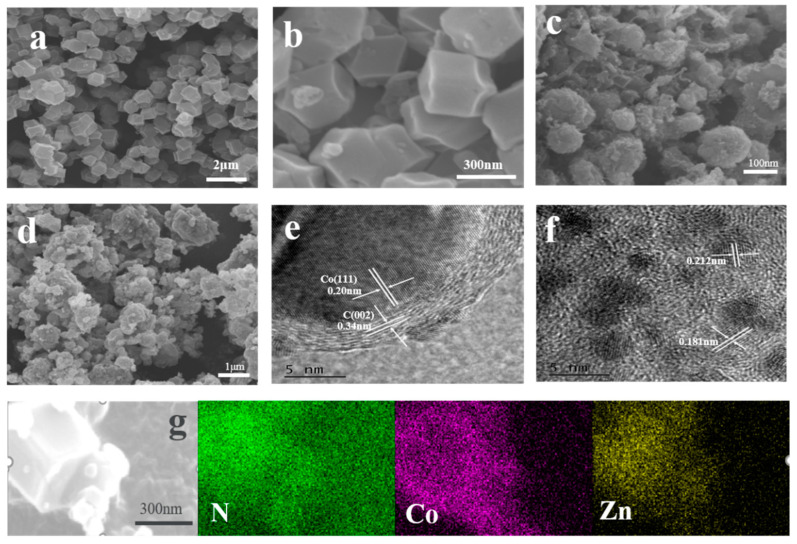
(**a**) SEM image of Co@ZIFs-1-6; (**b**) corresponding enlarged SEM image; (**c**) SEM image of MNC-1-6; (**d**) SEM image of PtNi/MNC-1-6; (**e**,**f**) TEM image of PtNi/MNC-1-6; (**g**) SEM image of Co@ZIFs-1-6 and its EDS image.

Scanning electron microscopy (SEM) and transmission electron microscopy (TEM) were applied to analyze the morphology of the catalyst, as shown in Figure 2a,b. It can be observed that the synthesized Co@ZIFs has a relatively uniform dodecahedral shape with a diameter of about 250 nm, indicating that most of the Co element was located on the surface of ZIF-8. As shown in Figure 2g, the outer surface of Co@ZIFs-1-6 is the Co element, and the inner part is the Zn element, which is basically consistent with the expectation. Meanwhile, it can be observed that the morphology of Co@ZIFs-1-3 does not achieve the expected results. It can be clearly seen in Figure S1a that its size is extremely uneven, which may be due to the excessive addition of Co elements, part of which form an independent ZIF-67 monomer, resulting in different sizes. After carbonization, the dodecahedral morphology of Co@ZIFs-1-6 is retained, but the surface becomes rough and the size is slightly reduced due to carbonization of organic bonds and atomic migration [4]. It is noteworthy that nanotubular structures in MNC-1-6 (Figure 2c and Supplementary Material S1c) were observed, possibly caused by the catalytic action of the metal Co on the surface of Co@ZIFs-1-6 during the carbonization process to form carbon nanotubes (CNTs) [5,16]. The generated CNTs are expected to be beneficial for the improvement of the specific surface area, catalytic activity, and stability of catalyst materials [16,17]. In addition, the numbers of the generated CNTs increased with the increases in the Co contents in the initial precursors. Figure 2d shows the morphology of PtNi/MNC-1-6. It can be seen that there are many deposited particles on the carbon carrier. In order to further explore the specific conditions of these sediments, electron transmission electron microscope (TEM) images were recorded, as shown in Figure 2e,f. The lattice spacing of 0.34 nm and 0.20 nm in Figure 2e corresponds to the C (002) plane and Co (111) plane and Co nanoparticles are coated with 8–10 layers of graphite-type carbon, which enhances the conductivity of the catalyst and the corrosion resistance of the metal particles coated by the carbon layer, thus improving the stability of the catalyst [18]. The lattice spacing of the particles shown in Figure 2f is 0.181 nm between the crystal plane of metal Pt (200) (0.196 nm) and metal Ni (200) (0.176 nm), and the lattice spacing of 0.212 nm is between the crystal plane of the corresponding metal Pt (111) (0.225 nm) and the metal Ni (111) (0.203 nm), confirming the formation of the PtNi alloys [19].

**Figure 3:**
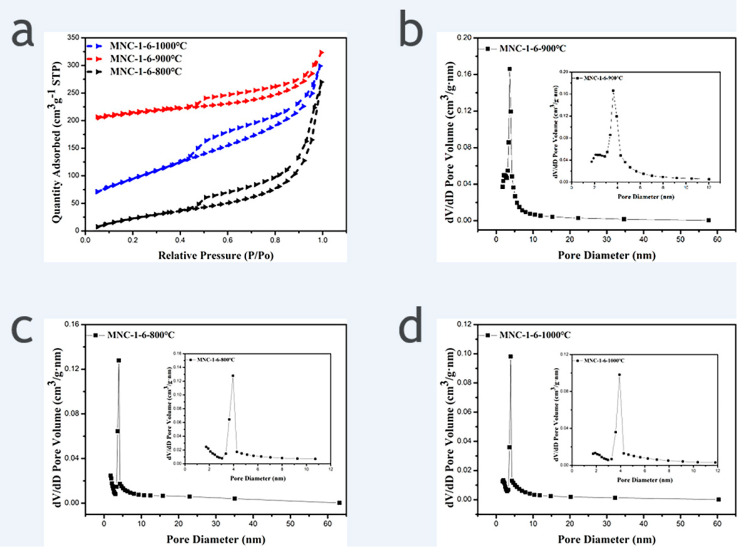
(**a**) N_2_ adsorption and desorption isotherms of MNC-1-6-800 °C, MNC-1-6-900 °C and MNC-1-6-1000 °C; (**b**–**d**) the corresponding pore size distribution curve (Inset: Enlarged view of the corresponding small size pore size distribution).

N_2_ adsorption and desorption isotherms were performed to determine the specific surface area and pore size distribution of samples. Figure 3 shows the N_2_ adsorption and desorption isotherms of MNC-1-6-800 °C, MNC-1-6-900 °C, and MNC-1-6-1000 °C and the corresponding pore size distribution curve. It is apparent that all the three recorded N_2_ adsorption and desorption isotherms exhibited H4 hysteresis loops, indicating that all the three carriers derived from different carbonization temperatures possess mesoporous structures, and the calculated pore sizes are mainly distributed at 3–5 nm. The derived specific surface area of MNC-1-6-800 °C, MNC-1-6-900 °C, and MNC-1-6-1000 °C are 251 m^2^/g, 343 m^2^/g and 119 m^2^/g, respectively. It is evident that the sample obtained from carbonization temperature of 900 °C exhibited the largest specific surface area among the three tested samples. This could suggest that the incomplete carbonization induced a less rough surface at 800 °C and the agglomeration of the derived carbon materials at 1000 °C are responsible for the relatively low specific surface area [20]. Evidently, the large specific surface area is beneficial to generation of active sites after the deposition of catalysts, which in turn can improve the utilization rate of the precious metal and the catalytic activity of the catalyst. Therefore, the carbonization temperature of 900 °C can be realized as the optimal condition for the synthesis of the catalyst support. 

**Figure 4:**
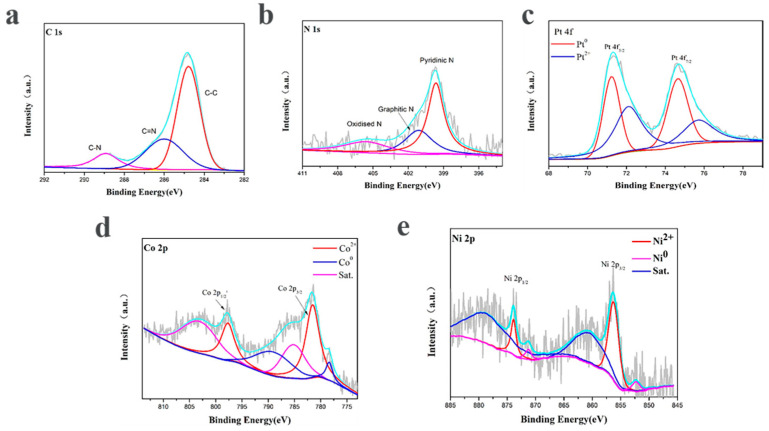
XPS spectra of PtNi/MNC-1-6: (**a**) C 1s; (**b**) N 1s; (**c**) Pt 4f; (**d**) Co 2p; (**e**) Ni 2p.

X-ray photoelectron spectroscopy was carried out to elucidate the composition and chemical states of the according elements near the surface of the PtNi/MNC-1-6. The XPS survey was corrected by the C 1s peak fixed at 284.8 eV. As shown in Figure 4a, a high-resolution C 1s peak is deconvoluted into C–C (284.8 eV), C–N (286.0 eV) and C–C=O bonds (289.8 eV), indicating that the sample contains nitrogen-doped carbon and graphite carbon [21].The corresponding N 1s peak can be deconvoluted into four peaks at 398.4 eV, 399.7 eV, 401.1 eV and 405.5 eV, corresponding to pyridine N, pyrrole N, graphite N and oxide N, respectively, among which pyrrole N and pyridine N are essential for the electrocatalytic activity of HER by interacting with H^+^ [16,21]. As shown in Figure 4d, Co 2p peak is deconvoluted and integrated into three pairs of peaks, among which the characteristic peaks at the binding energy of 778.4 eV and 789.1 eV are assigned to Co^0^, indicating the existence of metallic cobalt. The characteristic peaks at 781.7 eV and 797.7 eV correspond to Co 2p_3/2_ and Co 2p_1/2_, respectively, suggesting the presence of Co^2+^ in the sample, possibly due to the surface oxidation of the catalyst during the test [22]. The other peaks correspond to the satellite peaks of Co. Similarly, Figure 4e shows the deconcolution of the Ni 2p peak, the characteristic peaks of Ni^2+^ at 856.2 eV for Ni 2p_3/2_ and 873.8 eV for Ni 2P_1/2_ and the characteristic peaks for Ni^0^ at 852.3 eV and 871.2 eV. It is also found that the content of Ni^2+^ is slightly higher than Ni^0^, which is basically consistent with the results reported in the previous article [23,24]. In Figure 4c, the Pt 4f in the PtNi/MNC-1-6 sample contains two pairs of characteristic peaks, corresponding to the two valence states of the Pt element. The characteristic peaks at 71.2 eV and 74.6 eV are Pt 4f_7/2_ and Pt 4f_5/2_ belonging to Pt^0^, while the peaks at 72.1 eV and 75.7 eV are attributed to Pt^2+^ caused by surface oxidation [25,26].

### 2.2. Electrochemical Activity

**Figure 5:**
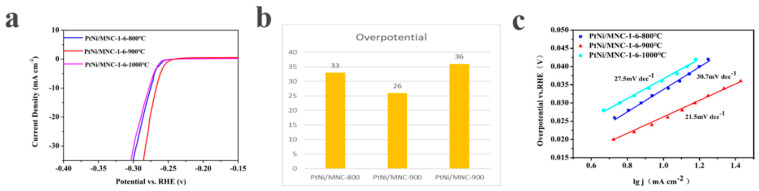
(**a**) HER polarization curve of the carrier-supported catalysts with different carbonization temperatures (polarization curves without i–R correction recorded in a 0.5 M H_2_SO_4_ solution at a sweep rate of 5 mV s^−1^); (**b**) the corresponding overpotential; (**c**) the fitted Tafel curve of the corresponding polarization curve.

The influence of the carbonization temperature on the performance of the catalyst was investigated through the polarization curves of samples derived from different carbonization temperatures. As shown in Figure 5a,b, the required overpotentials for reaching a current density of 10 mA cm^−2^ are 33 mV, 26 mV and 36 mV for PtNi/MNC-1-6-800 °C, PtNi/MNC-1-6-900 °C and PtNi/MNC-1-6-1000 °C, respectively, suggesting that PtNi/MNC-1-6-900 °C exhibits the best performance among the tested samples, due to its high surface area, which enables the uniform distribution of the PtNi alloy on the surface of support. In addition, the relatively low carbonization temperature of 800 °C leads to the low content of graphitized carbon, which accordingly results in low conductivity and the catalytic performance [27]. Tafel curves derived from polarization curves were plotted in Figure 5c to explore the kinetics of the HER process for the three samples. It was observed that the Tafel slope of PtNi/MNC-1-6-900 °C is only 21.5 mV dec^−1^, much lower than 30.7 mV dec^−1^ for PtNi/MNC-1-6-800 °C and 27.5 mV dec^−1^ for PtNi/MNC-1-6-1000 °C, indicating that the current density of sample PtNi/MNC-1-6-900 °C increased much faster with the increase in overpotential compared to the other two samples, implying the fast electrochemical reaction kinetics [23].

**Figure 6:**
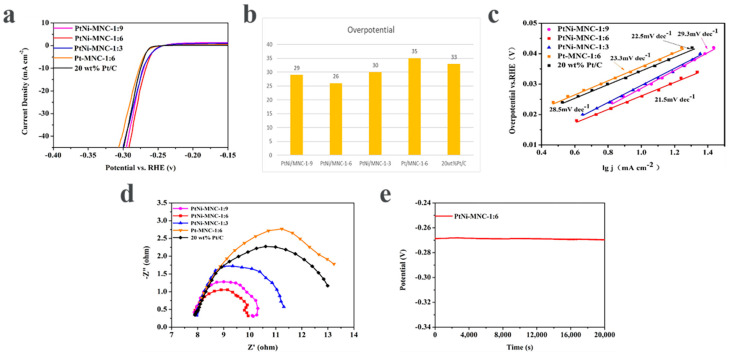
(**a**) Polarization curves of various catalysts (polarization curves without i–R correction recorded in a 0.5 M H_2_SO_4_ solution at a sweep rate of 5 mV s^−1^); (**b**) corresponding overpotential image of catalysts; (**c**) the fitted Tafel curve of the corresponding polarization curve; (**d**) electrochemical impedance spectroscopy of various catalysts and 20 wt% Pt/C samples; (**e**) the chronopotential curve of PtNi/MNC-1-6.

The influence of the introduced Ni to the Pt catalyst on the HER performance was also investigated. As shown in Figure 6a,b, the sample of PtNi/MNC-1-6 only needs the overpotential of 26 mV to reach a current density 10 mA cm^−2^, superior to Pt/MNC-1-6 (35 mV). In addition, the advantage of the nickel-containing sample is also reflected by the Tafel curves. As can be seen from Figure 6c, the Tafel slope of sample PtNi/MNC-1-6 is 21.5 mV dec^−1^, which is also lower than that of Pt/MNC-1-6 (23.3 mV dec^−1^). It can also be clearly seen from the impedance spectra that the diameter of the impedance semicircle for sample PtNi/MNC-1-6 is much smaller than that for Pt/MNC-1-6, indicating that sample PtNi/MNC-1-6 has a much smaller charge transfer resistance (Rct) than Pt/MNC-1-6 does. The results demonstrate that the introduced transition metal Ni can adjust the intrinsic electronic structure of Pt and improve the catalytic activity [8,28]. In Table S1, we have listed summary of various PtNi alloys electrocatalysts for HER performance in 0.5 M H_2_SO_4_.

The influence of the ratio of Co to Zn in the support on the performance of the catalyst was explored. As shown in Figure 6a,b, to reach a current density 10 mA cm^−2^, the overpotential of sample PtNi/MNC-1-6 is 26 mV, about 4 mV and 3 mV lower than those of sample PtNi/MNC-1-3 (30 mV) and sample PtNi/MNC-1-9 (29 mV), respectively. In addition, the overpotentials of all the three samples are lower than that of commercial 20 wt% Pt/C (33 mV), possibly attributed to the Pt–Ni interaction and PtNi-alloy-support interaction. The best performance of sample PtNi/MNC-1-6 among all the tested samples is mainly attributed to the appropriate Co and Zn mass ratio induced porous morphology and the amount of generated CNTs as discussed in the above section.

The derived Tafel curve from the polarization curve is applied to elucidate the kinetics of the HER process. Under acidic conditions, hydrogen evolution reaction is generally analyzed by two mechanisms in three steps [29,30]. The three steps are the Volmer, Heyrovsky, and Tafel steps, and the corresponding Tafel slope is 120 mV dec^−1^, 40 mV dec^−1^ and 30 mV dec^−1^, respectively. The two mechanisms are the Volmer–Tafel mechanism and Volmer–Heyrovsky mechanism. The reaction of these three steps is as follows:

H_3_O^+^ + e^−^ → H* + H_2_O (Volmer) (1)

H* + H_3_O^+^ + e^−^ → H_2_ + H_2_O (Heyrovsky) (2)

H* + H* → H_2_ (Tafel) (3)

H*: H of adsorption state.

The Tafel slopes of PtNi/MNC-1-3, PtNi/MNC-1-6 and PtNi/MNC-1-9 are 28.5 mV dec^−1^, 21.5 mV dec^−1^ and 29.3 mV dec^−1^, respectively. It can be observed that the Tafel slope of sample PtNi/MNC-1-6 is lower than that of 20 wt% Pt/C (22.5 mV dec^−1^), indicating that PtNi/MNC-1-6 exhibited faster electrochemical kinetics than commercial Pt/C. In addition, the HER process using the designed materials in this work follows the Volmer–Tafel mechanism.

Electrochemical impedance spectroscopy was applied to analyze the reaction kinetics of each catalyst. As shown in Figure 6d, the charge transfer resistance of PtNi/MNC-1-6 is much lower than that of PtNi/MNC-1-3, PtNi/MNC-1-9 and 20 wt% of Pt/C, indicating that PtNi/MNC-1-6 has lower charge transfer resistance and faster electrode kinetics [28]. The low charge transfer resistance is mainly due to appropriate carbonization temperatures and the strong interaction between PtNi and the support [31]. The chronopotentiometry technique was used to evaluate the stability of the catalyst. As shown in Figure 6e, the potential of the catalyst remained almost unchanged after continuous operation at the potential corresponding to 10 mA cm^−2^ for 20,000 s, demonstrating the great stability of PtNi/MNC-1-6.

## 3. Conclusions

In this work, the PtNi alloy was loaded on ZIF-derived carbon support as a catalyst for hydrogen evolution reaction and the influence of synthetic conditions and catalyst composition on the performance of the catalyst was studied. The catalyst was characterized and analyzed by physical characterization methods, such as XRD, SEM, TEM, BET, XPS, and LSV, EIS, it, v-t, etc. The optimal sample PtNi/MNC-1-6 (Pt content 8.1%) requires only 26 mV overpotential to reach a current density of 10 mA cm^−2^ with a small Tafel slope of 21.5 mV dec^−1^. Moreover, the sample shows good stability. The excellent performance of the synthesized sample mainly benefits from the following points. (1) The proper Co and Zn ratio and carbonization temperature of the carrier provide good conductivity and a large specific surface area, which is conducive to the full dispersion of precious metals and improve the utilization rate of precious metals. (2) The synergistic effect of metal Pt and Ni improves the catalytic activity of the catalyst. (3) The interaction between the PtNi alloy and carbon support is beneficial to the rapid transfer of electrons.

## Figures and Tables

**Table 1:** Content test of each element in the PtNi/MNC-1-6 sample.

Sample	Measured Element	Mass Fraction (wt%)
PtNi/MNC-1-6	Pt	8.1
	Ni Co Zn	2.9 12 0.022

## Data Availability

Not applicable.

## Supplementary Materials

The following are available online: Experimental section; Figure S1 and Table S1.

## Sample Availability

Samples are available from the authors.
