# Supplementary material for: PtNi Alloy Coated in Porous Nitrogen-Doped Carbon as Highly Efficient Catalysts for Hydrogen Evolution Reactions
Source: Molecules. 2022 Jan 14;27(2):499. doi: 10.3390/molecules27020499 (PMC8778313; doi:10.3390/molecules27020499)
Supplement: Supplementary file 1 [file molecules-27-00499-s001.zip › molecules-1546584-supplementary.pdf]

PtNi alloy coated in porous nitrogen-doped carbon as highly efficient catalysts for hydrogen evolution reaction

Xuyan Song[ab], Yunlu He[a], Bo Wang[a], Sanwen Peng[b], Lin Tong[a], Qiang Liu[a], Jun Yu*[c], Haolin Tang*[c]

a China Tobacco Hubei Industrial Co., Ltd., Wuhan, 430051, China

b China Tobacco Hubei Industrial Cigarette Materials Co., Ltd., Wuhan, 430051, China

c State Key Laboratory of Advanced Technology for Materials Synthesis and Processing, Wuhan University of Technology, Wuhan, 430070, China

*Corresponding author E-mail: yujun@whut.edu.cn

Experimental section

Materials

methanol (≥99.5%), benzyl alcohol (≥99.0%), isopropanol (≥99.7%), Cobalt nitrate hexahydrate (≥99%), zinc nitrate hexahydrate (≥99%), citric acid monohydrate (≥99.5%), concentrated sulfuric acid (95-98 wt%) were purchased from Sinopharm Chemical Reagent Co., Ltd. (Shanghai, China). 2-methylimidazole (98%), chloroplatinic acid hexahydrate (Ptr≥37%), nickel acetylacetonate (95%) were obtained from Alladin Ltd. (China). Commercial Pt/C (Pt:20 wt%) was purchased from Johnson Matthey and Nafion (5 wt%) was acquired from DuPont separately. All the reagents were used as received without further treatment.

Characterization

Powder X-ray diffraction (XRD) patterns were collected through Bruker D8-Advance X-ray diffractometer using Cu ka radiation (λ=1.5406 Å). Talos-F200S field emission transmission electron microscopy (TEM) and JEM-7500 F field emission scanning electron microscopy (FE-SEM) were used to study morphology and structure of the catalysts. The mass fraction of Pt, Ni, Co and Zn was determined by inductively coupled plasma-optical emission spectroscopy (ICP-OES, Prodigy 7). Chemical state of samples showing in X-ray photoelectron spectroscopy (XPS) images were performed on ESCALAB-250Xi spectrometer equipped with monochromatic Al Ka radiation.

ASAP 2020 M fully automatic specific surface area and porosity analyzer was applied to analyze N_2 adsorption-desorption isotherm and the sample was activated in vacuum at 623 K for 6 h.

Preparation of ZIF-8

Solutions A and B were first synthesized. Solution A: 3.72g Zn $(NO_3)_2·6H_2O$ was ultrasonically dispersed in 40 mL methanol for 3 min. Solution B: 4.1g 2-methyimidazole was dissolved in 40 mL methanol to form uniform solution quickly. Then, solution A was added into solution B and the mixed solution kept stirring for 15 min. After that, the solution was stirred at room temperature for 4 hours. Finally, the mixture was centrifuged and washed with methanol for several times. And the ZIF-8 nanoparticles were obtained after being dried in vacuum at 80°C for 12h.

Synthesis of doped ZIFs (Co@ZIFs)

Solutions A and B were first synthesized. Solution A: 200mg prepared ZIF-8 and 600mg Co $(NO_3)_2·6H_2O$ was ultrasonically dispersed in 60 mL methanol for 10 min. Solution B: 1300mg 2-methyimidazole was dissolved in 20 mL methanol to form uniform solution quickly. Then, solution B was added into solution A and the mixed solution was ultrasonically dispersed for 15 min. After that, the purple solution was stirred at room temperature for 24 hours. Finally, the mixture was centrifuged and washed with methanol for several times. And the Co@ZIFs nanoparticles were obtained after being dried in vacuum at 80°C for 12h. Under the same conditions, changing Co

$(NO_3)_2 \cdot 6H_2O$ and 2-methylimidazole into 300mg and 650mg (900mg and 1950mg), and the product is Co@ZIFs-1-9 (Co@ZIFs-1-3).

Preparation of MNC-900°C/800°C/1000°C

A certain of Co@ZIFs powder was put into a tube furnace and carbonized at 900 °C (or 800 °C, 1000 °C) in high purity N_2 atmosphere for 2.5h with a heating rate of 5°C min^{-1}. The as-received product was named as MNC-900°C (or MCN-800 °C, MCN-1000 °C) was gained by natural cooling to room temperature. Name the carbonized products of Co@ZIFs-1-3, Co@ZIFs-1-6 and Co@ZIFs-1-9 as MNC-1-3, MNC-1-6 and MNC-1-9, respectively

Synthesis of PtNi/MNC-x-y.

MNC (500mg), citric acid (100mg), nickel acetylacetonate (13mg) and 3ml $H_2Cl_6Pt \cdot 6H_2O$ (19.31 mmol L^{-1}) solution 19.31 mmol L^{-1} were added into 40 mL benzene methanol solution. The mixed solution was evenly dispersed by ultrasound for 1h, and then was bathed in oil for 5 hours at 160 ° C, and cooled to room temperature naturally. Finally, the powder was put into a tube furnace and calcined in 5% H_2/Ar flow at 200°C for 2 h with a heating rate of 5°C min^{-1}. The final product is named as PtNi/MNC-x-y..

Electrochemical measurements

Electrochemical tests were operated in 0.5M H_2SO_4 solution on the CHI660 electrochemical workstation using a typical three electrode system at room temperature. None of the tests were iR compensated. Glassy-carbon disk, graphite rod and saturated

calomel electrode (SCE) were used as work, counter and reference electrodes. To obtain homogeneous catalyst ink, 5 mg sample was ultrasonically dispersed in centrifugal tube containing 980 μl isopropanol and 20 μl Nafion solution (5 wt%) for 15 min. After that, 4 μl catalyst suspension (0.283 mg cm^{-2}) was dropped onto the pre-prepared 3 mm diameter glassy carbon disk. For comparison, 20% Pt/C and other sample were treated using the same way. Before the HER measurement, the working electrode kept cycling for 30 times at 100 mV s^{-1} to activate the catalyst and obtain stable curve. The linear sweep voltammetry was performed out between -0.1 V and -0.3 V vs. SCE at 5 mV/s. The frequency range for electrochemical impedance spectroscopy (EIS) was from 100 kHz to 0.01 Hz with an amplitude of 5 mV at -0.268 V vs. SCE (0.5M H_2SO_4).The durability of PtNi/MNC-1-6 was evaluated at a current density of 10 mA cm^{-2} using chronopotentiometry.

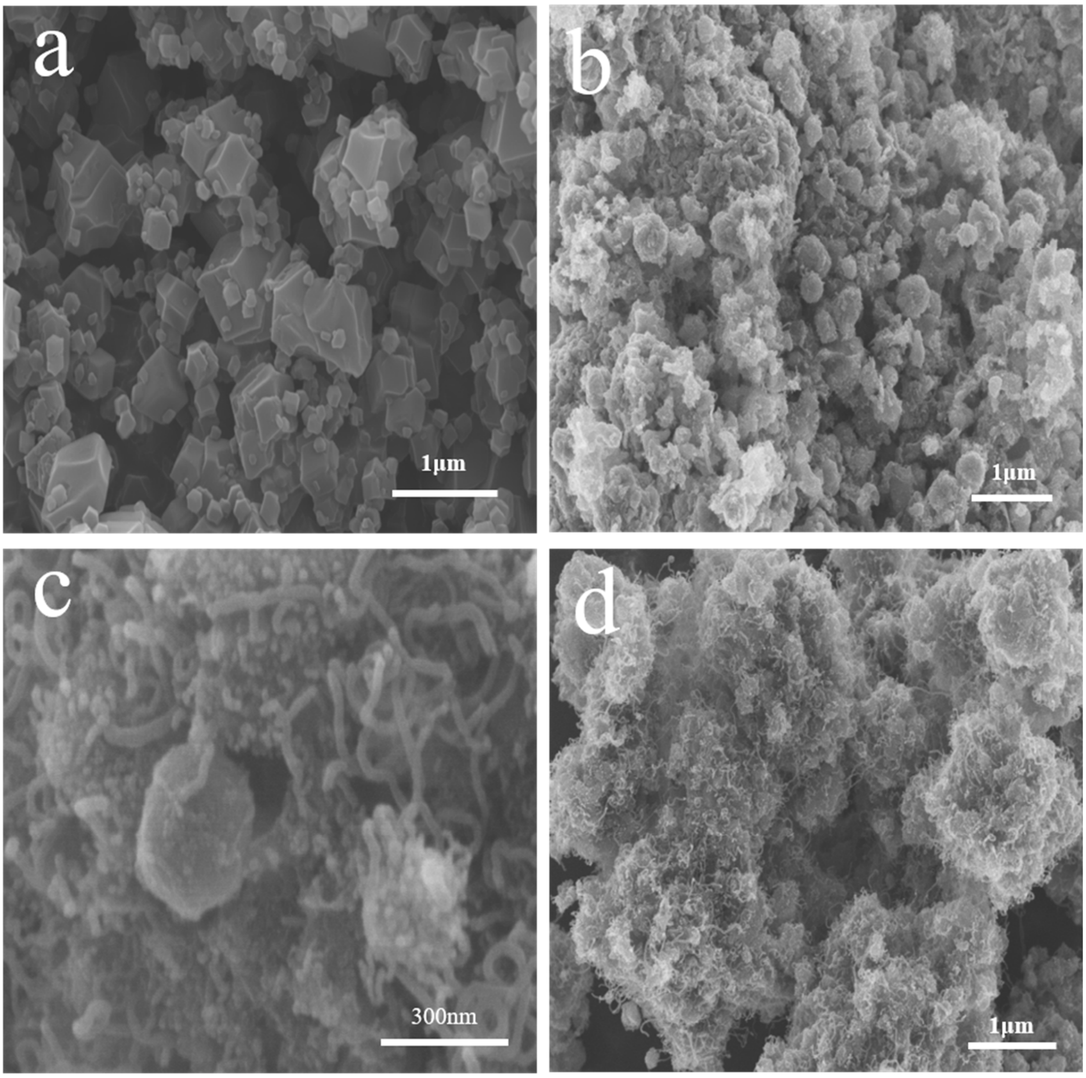

Figure S1 SEM images of (a, d) Co@ZIFs-1-3; (b) MNC-1-9; (c) MNC-1-6; (d) MNC-1-3

Table S1 Summary of various PtNi **alloys** electrocatalysts for HER performance in 0.5M H_2SO_4

Material	Electrolyte	η_{10} (mV)	Tafel slope (mV dec^{-1})	Rct (Ω)	Reference
**PtNi/MNC-1-6**	0.5 M H_2SO_4	26	21.5	2	This work
**PtNi/10PG CNCs,**	0.5 M H_2SO_4	31	18	12.99	[1]
**PtNi/NDs**	0.5 M H_2SO_4	26	22		[2]
**$Pt_{3.21}Ni@Ti_3C_2$**	0.5 M H_2SO_4	19	13.3		[3]
**$PtNi/Ni_4B_3$**	0.5 M H_2SO_4	25.6	25.1	4.1	[4]
**$PtNi_2$@CNS-600**	0.5 M H_2SO_4	68	35		[5]
**PtNi/CNFs**	0.5 M H_2SO_4	34	31	34	[6]
**PtNi/rGO(N)**	0.5 M H_2SO_4	98	43		[7]

1. Yang, J. T.; Ning, G. Q.; Yu, L.; Wang, Y.; Luan, C. L.; Fan, A. X.; Zhang, X.; Liu, Y. J.; Dong, Y.; Dai, X. P., Morphology controllable synthesis of PtNi concave nanocubes enclosed by high-index facets supported on porous graphene for enhanced hydrogen evolution reaction. *Journal of Materials Chemistry A* **2019,** *7* (30), 17790-17796.
2. Yan, Y.; Zhang, R. Z.; Yu, Y. D.; Sun, Z. M.; Che, R. C.; Wei, B.; LaGrow, A. P.; Wang, Z. C.; Zhou, W., Interfacial optimization of PtNi octahedrons@Ti3C2MXene with enhanced alkaline hydrogen evolution activity and stability. *Applied Catalysis B-Environmental* **2021,** *291*.
3. Jiang, Y.; Wu, X.; Yan, Y.; Luo, S.; Li, X.; Huang, J.; Zhang, H.; Yang, D., Coupling PtNi Ultrathin Nanowires with MXenes for Boosting Electrocatalytic Hydrogen Evolution in Both Acidic and Alkaline Solutions. *Small* **2019,** *15* (12).
4. Yuan, M. Y.; Xu, H.; Wang, C.; Wang, Y.; Wang, Y.; Wang, X. M.; Du, Y. K., PtM/MxBy (M=Ni, Co, Fe) Heterostructured Nanobundles as Advanced Electrocatalyst for Hydrogen Evolution Reaction. *Chemistry-a European Journal* **2021,** *27* (50), 12851-12856.
5. Li, J.; Liu, L.; Ai, Y.; Hu, Z.; Xie, L.; Bao, H.; Wu, J.; Tian, H.; Guo, R.; Ren, S.; Xu, W.; Sun, H.; Zhang, G.; Liang, Q., Facile and Large-Scale Fabrication of Sub-3 nm PtNi Nanoparticles Supported on Porous Carbon Sheet: A Bifunctional Material for the Hydrogen Evolution Reaction and Hydrogenation. *Chemistry-a European Journal* **2019,** *25* (29), 7191-7200.
6. Chen, J. W.; Wang, J.; Chen, J. D.; Wang, L. N., A bifunctional electrocatalyst of PtNi nanoparticles immobilized on three-dimensional carbon nanofiber mats for efficient and stable water

splitting in both acid and basic media. *Journal of Materials Science* **2017,** *52* (22), 13064-13077.
7. Bao, J. H.; Wang, J. Q.; Zhou, Y. M.; Hu, Y. J.; Zhang, Z. W.; Li, T. F.; Xue, Y.; Guo, C.; Zhang, Y. W., Anchoring ultrafine PtNi nanoparticles on N-doped graphene for highly efficient hydrogen evolution reaction. *Catal. Sci. Technol.* **2019,** *9* (18), 4961-4969.
